# The effect of hydroalcoholic extract of *Psidium guajava L.* on experimentally induced oral mucosal wound in rat

**DOI:** 10.1186/s12906-022-03655-5

**Published:** 2022-07-29

**Authors:** Faezeh Ghaderi, Elham Ebrahimi, Fatemeh Sari Aslani, Omid Koohi-Hosseinabadi, Farhad Koohpeyma, Cambyz Irajie, Nader Tanideh, Aida Iraji

**Affiliations:** 1 Department of Pediatrics Dentistry, Shiraz University of Medical Sciences, Shiraz, Iran; 2 Member of Student Research Committee, Shiraz University of Medical Sciences, Shiraz, Iran; 3 Department of Pediatric Dentistry, Faculty of Dentistry, Shiraz branch, Islamic Azad University, Shiraz, Iran; 4 Molecular Dermatology Research Center, Shiraz University of Medical Sciences, Shiraz, Iran; 5 Department of Pathology, Shiraz University of Medical Sciences, Shiraz, Iran; 6 Laparoscopy Research Center, Shiraz University of Medical Sciences, Shiraz, Iran; 7 Present Address: Central Research Laboratory, Shiraz University of Medical Sciences, Shiraz, Iran; 8 Endocrinology and Metabolism Research Center, Shiraz University of Medical Sciences, Shiraz, Iran; 9 Department of Medical Biotechnology, School of Advanced Medical Sciences and Technologies, Shiraz University of Medical Sciences, Shiraz, Iran; 10 Stem Cells Technology Research Center, Shiraz University of Medical Sciences, P. O. Box: 7134845794, Shiraz, Iran; 11 Department of Pharmacology, Shiraz Medical School, Shiraz University of Medical Sciences, Shiraz, Iran

**Keywords:** Oral mucositis, Oxidative stress, Natural product, Inflammation, Oral health

## Abstract

**Background:**

The aim of this study was to evaluate the biological effects of hydroalcoholic extract of *Psidium guajava L* leaves and phenytoin as a standard agent on the induced oral mucosal wound.

**Methods:**

Hundred seventy Sprague Dawley rats were grouped in 5 clusters randomly. Oral mucosal wounds were induced in all rats except for the control group. Phenytoin and guajava leaf extract were used as a mouthwash. Twelve rats from the 5 groups were euthanized on day 7^th^ and 10^th^, and 10 rats from each group were sacrificed on the 14^th^ day. Interleukin-6 and total antioxidant capacity were determined in the serum. The tissues were evaluated for pathological and stereological assessments. Phytochemical analyses were performed on the hydroalcoholic extract of *Psidium guajava L* to determine the antioxidant potency.

**Results:**

Total phenolic content test and DPPH analysis demonstrated the high potential of antioxidant capacity of *Psidium guajava L.* Decreasing IL-6 and increasing TAC were seen in the guajava hydroalcoholic extract and phenytoin groups. The difference of IL-6 between the wound treated guajava group and the wounded group was significant. The wound treated guajava group and wound treated phenytoin group on the 14^th^ day increased the number of fibroblast cells and volume density of sub-mucosae effectively to the same thickness to be considered as a healed sub-mucosae layer. The volume density of the epithelium changes showed statistically significant different responses based on gender.

**Conclusion:**

In conclusion, hydroalcoholic extract of *Psidium guajava L* leaves might exert theraputic effects on oral mucositis.

**Supplementary Information:**

The online version contains supplementary material available at 10.1186/s12906-022-03655-5.

## Background

Oral mucositis (OM) is a frequent inflammatory complication of cancer chemo- or radiotherapy that is characterized by pain, erythema, and formation of ulcers. OM patients have difficulty in speaking, swallowing, and alimentation, which contribute to weakness in daily functions and wellbeing [1]. Currently, OM treatment is based on symptomatic care due to the lack of effective treatments; to manage OM complications, experts have suggested different therapeutic agents including good oral hygiene and anti-inflammatory agents [2]. The oral environment has specific physiologic conditions that alter the normal healing process; therefore, the high level of oral hygiene is an important factor in the ulcer site [3]. Different pharmacological agents were suggested for oral traumatic wound healing, such as mixed topical antimicrobial agents and steroids which reduced the accumulation of plaque, post-operative pain, and swelling [4].

Phenytoin (C_15_H_12_N_2_O_2_) is an insoluble weak acid, usually administered as salt to improve solubility [5]. Phenytoin is a major anti-epileptic and anticonvulsant drug possessing proliferative activities via accelerating the healing process in the epithelium [6]. The common side effect of phenytoin is the development of fibrous tissue overgrowth of the gingiva which resulted in acceleration in wound healing [7]. The effects of phenytoin on the wound healing process have been evaluated in different studies; the biological data confirmed that phenytoin had wound-healing actions which contributed to the increase in the proliferation of fibroblasts, improvement in the deposition of collagen, and enhancement of granulation tissue formation. Besides, the decrease in the action of collagenase and bacterial contamination in the wounds was also reported during the consumption of phenytoin [6].

Topical steroids and/or local anesthetics are not recommended on the inflammated oral wounds as they suppress the immune functions, or might mask other severe symptoms. Attempts are now focusing on developing novel substances with minimal side effects with profound therapeutic effects [8, 9].

Recently, research on phytochemicals and their effects on public health has been intensively increased. In particular, research has focused on finding powerful natural antioxidants with wound healing properties. *Psidium guajava L.*, popularly known as guajava, is growing wild in the torrid zone and subtropics [10]. *Psidium guajava L* is cultivated in Taiwan, South American countries, Brazil, Colombia, Mexico, Venezuela, and southeast areas of Iran [11].

Guajava flowers and green leaves contain high amounts of α-pinene, β-pinene limonene, tannins, flavonoids, flavonoids, rutin, saponins as well as quercetin, luteolin, guajavolide, and kaempferol [12]. The pharmacological studies conducted on *P. guajava* indicated the immense potential of this plant in the treatment of diarrhea, wounds, acne, dental plaque, malaria, allergies, coughs, diabetes, and cardiovascular disorder [13]. Recently, the herbal oral gel containing extracts of *P. guajava Linn* leaves and *Curcuma longa Linn rhizomes* were used to treat mouth ulcers. The results showed that the mentioned extract had significant antimicrobial activities which are important to develop anti-ulcer agents [14]. The antifungal activity of the gel was confirmed via reducing the zone of inhibition against *Candida albicans* to 24 mm compared to positive control with 27 mm [15]. Also, other studies showed strong antibacterial activity against the gram-positive and the gram-negative as well as antifungal potency, especially against *C. albicans* [16]. On the other hand, the high antioxidant and anti-inflammatory potency of guajava was reported in various studies that were related to a high amount of polyphenolic compounds. Biological data depicted significant anti-inflammatory activity of guajava against acute, subacute, and chronic inflammation [17, 18] and promising reduction of iNOS, COX-2, and NF-κB [19]. Also, the high potency and efficacy of guajava as an anti-ulcer treatment is attracting attention nowadays [20–22].

Common causes of mouth ulcers are nutritional deficiencies (especially Fe, B12, and C), poor oral hygiene, and infections. On the other hand, microbial biofilms, overexpression of inflammatory cytokines, and high levels of reactive oxygen/nitrogen species (ROS, RNS) activate the inflammatory phase, inhibiting progression to the proliferation and reepithelialization stages [23–25]. It was proposed that for effective treatment, multi‐therapeutic strategies should be applied to treat wound ulcers. In this regard, growing pieces of evidence suggested that guajava could be an ideal multi-therapy agent. The key features propose that guajava has a high amount of vitamin C even more than citrus (80 mg of vitamin C in 100 g of fruit), vitamin A and other important minerals including K, Ca, Fe and Zn [26, 27]; secondly, it is a powerful antimicrobial agent which reduces the complication of ulcer [28, 29]. Third, guajava is a powerful antioxidant and anti-inflammatory natural remedy.

The aim of the present study was to evaluate the therapeutic effect of guava as a source of natural antioxidant and bioactive compounds on the wound healing process on the induced oral mucosal wound in rats. Also, the total phenolic compound (TPC) and antioxidant potential of the extract were measured. Phenytoin was used as a reference compound for wound healing activity.

## Methods

### Ethical considerations

Ethical approval was confirmed by the Animal Care Committee of Shiraz University of Medical Sciences (IR.SUMS.REC 0.1395. S928). The authors followed up all institutional and international guidelines for animal care and use during this study under the supervision of the ethical committee of Shiraz University of Medical Sciences. The Animal Research Reporting in Vivo Experiments guidelines (ARRIVE) and international legislation, as well as AVMA Guidelines for the Euthanasia of Animals, were also followed up.

### Animal study and housing

The experiments were carried out on 170 Sprague Dawley rats with equal utility in both sexes (weighing 220 ± 20 g, 10–12 weeks aged, 12-h light/ 12-h dark, the temperature of 23 ± 1 °C, 55% ± 5 humidity). Animals were obtained from the Laboratory Animal Center of Shiraz University of Medical Sciences. They were fed with a standard diet and water ad libitum [30]. In the normal condition, the daily rat food consumption was around 7 to 10 g. Rats were randomly divided into 5 groups (34 animals in each group) as follows:

- Group 1: control group (no induced wound)
- Group 2: wound + no intervention
- Group 3: wound + normal saline (W + N.S), 0.9% mouthwash, every day.
- Group 4: wound + phenytoin suspension (W + P), 30 mg /5 ml mouthwash, every day.
- Group 5: wound + hydroalcoholic extract of the guajava leaves (W + G), 10% mouthwash, every day.

### Induction of oral buccal mucosa wound in rats

**Fig. 1:**
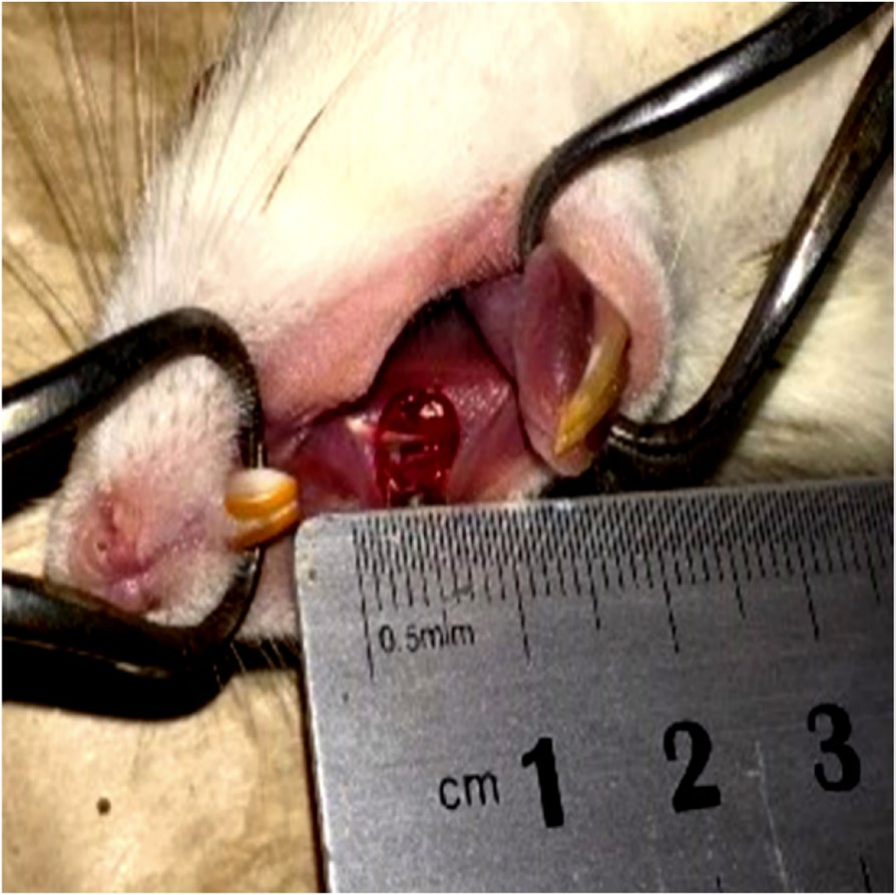
Induced oral buccal mucosa wound

The rats were anesthetized with intramuscular injection of ketamine hydrochloride 10% (100 mg/kg) and xylazine 2% (10 mg/kg). Excising the soft tissue of oral buccal mucosa was done with a punch (Trephine, circular scalpel) of 5 mm diameter in all rats, except for group 1[31]. (Fig. 1). Rats were euthanized with the rapid and humane method using a 70% volume displacement rate of CO_2_ increases around 100% in the induction chamber.

### Preparation of hydroalcoholic extracts of Guajava leaves

In this work, the plant material was collected from the city of Chabahar, Sistan and Baluchestan Province, Iran in 2019 (southeast of Iran) in large quantities to reduce the variety of components in the plant. A voucher specimen was deposited in the herbarium of the Faculty of Pharmacy, Shiraz University of Medical Sciences, Shiraz, Iran; then, the plants were identified (Voucher No. 771). The leaves were air-dried at ambient temperature. The hydro-ethanolic extract was prepared by adding 100 g of dried leaves to 500 ml hydro-ethanol solution (70% ethanol) at room temperature, through fractionated percolation, and then the final mixture was filtered. The obtained extract after filtration was concentrated in vacuum using a rotary flash evaporator [32]. There was a net yield of 24 g of a concentrated extract. The dried extract was then diluted to 10% solution with distilled water for mouth washing.

### Phenytoin suspension and normal saline

Suspension of phenytoin (30 mg/ ml, Alhavi Co. Iran) and normal saline 0.9%, (Tehran-Daru Co. Iran) were used for mouth washing. Mouth washing was done for each rat by using 5 ml of the specified solution for 2 min over the wound surface twice daily. 12 rats (Female = 6 + Male = 6) from every 5 groups were euthanized ethically on days 7^th^ and 10^th^ and 10 rats from each group on day 14^th^. Serum was obtained via cardiocentesis for assessments of IL-6 and TAC on determined days. The sample tissues were sectioned into pieces (4 µm and 25 µm in thickness) and stained with hematoxylin and eosin (H&E) for histopathological and stereological evaluations.

### Antioxidant activity of the extract based on DPPH assay

Assay for the scavenging of stable free radical 1,1-diphenyl-2-picrylhydrazyl (DPPH) was done according to Brand-Williams et al.’s (1995) method with slight modifications [33, 34]. Quercetin was used as a positive control, and the antioxidant activity of the extract was expressed as IC_50_. The experiment was repeated 3 times and the IC_50_ value was calculated by best-fit equations using the Curve Expert statistical program [35–37].

### Determination of total phenolic content (TPC)

The TPC of Guajava extract was determined using the Foline–Ciocalteu procedure, as described previously with minor modifications [38, 39]. The concentration of the total phenolic was expressed as milligrams of gallic acid equivalents per gram of dry extract (mg of GA/g of dE) [3, 4].

### Measurement of IL-6 and TAC

An Ebiosience kit (Biosource, USA) was obtained and the serum level of inflammatory factor interleukin-6 (IL-6) was determined by enzyme-linked immunosorbent assay (ELISA). Besides, the total antioxidant capacity (TAC) in the serum was evaluated by colorimetric method using a commercial kit (Cayman, USA) based on a previous study [40].

### Stereological evaluation

**Fig. 2:**
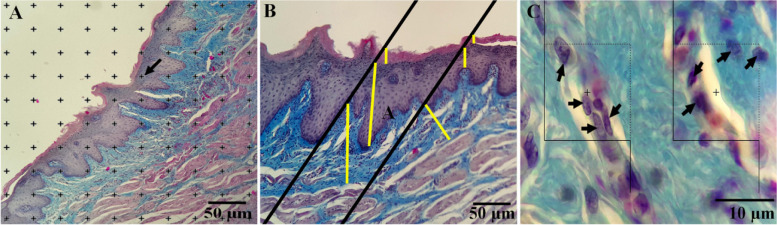
**A** Point counting method, ((The accepted points which hit the right upper corner of each cross of the targeted islets were counted (arrow)). **B** The orthogonal intercept method for measurement of the thickness of the different layers **C** Optical dissector method ((In this method, the cells were counted which were placed inside or on the accepted line (Dotted lines) and not rejected lines (lower and left borders))

Stereological evaluation of buccal mucosal tissue of the wounded area was performed according to previously reported procedures [41]. Briefly, for analyzing quantitative histomorphometric images the software (Stereo-Lite, SUMS, Shiraz, Iran) was used. On the other hand, to capture the microscopy images a video-microscope system composed of a microscope (Nikon E-200, Japan), a digital camera (Samsung SCB- 2000 P, Korea), and a personal computer were used. Finally,the images were processed using Adobe Photoshop CC (Adobe System) to enhance the resolution of the images. To estimate the mean height of the layers (epithelium, whole mucosa), the thickness was measured vertically at the meeting points of the lines and mucosa (Fig. 2A and B).

The numerical density of the fibroblasts was also measured using the “optical disector” method (Fig. 2C).

### Histopathological evaluation

The histological evaluation was based on the degree of tissue healing using a 4-point scoring system ranging from grade 0–3 as Lima’s method [42]:

- Score 0: normal epithelium and connective tissue without vasodilatation; absence of discreet cellular infiltration; absence of hemorrhagic areas, ulcerations or abscesses.
- Score 1: discreet vascular ingurgitation, re-epithelialization, discreet inflammatory infiltration with mononuclear prevalence; absence of hemorrhagic areas, edema, ulcerations or abscesses.
- Score 2: moderate vascular ingurgitation, areas of hydropic epithelial degeneration, inflammatory infiltration with neutrophil prevalence, presence of hemorrhagic areas, edema and eventual ulcerations, absence of abscesses.
- Score 3: severe vascular ingurgitation and dilatation, inflammatory infiltration with neutrophil prevalence, presence of hemorrhagic areas, edema, and extensive ulcerations and abscesses.

### Statistical analysis

All the statistical analyses were performed using the SPSS statistical software (v. 25). For all the above methods, the results were expressed as mean ± standard error. Statistical analysis was done using parametric (multiple comparison test) or non-parametric statistics based on the normality test. Then, the data were analyzed using the Mann–Whitney U test. Besides, differences were considered significant at *P* < 0.05.

## Results

### Phytochemical analyses

#### Antioxidant activity

The stable free radical DPPH was used to estimate the activity of Guajava extract as a source of antioxidants to decrease and inactivate the initial DPPH radical concentration to 50% in 30 min (IC_50_ ± SD). The DPPH radical scavenging activity expressed as IC_50_ was 172 ± 3.592 ng/ml. It seems that Guajava extract was found effective to prevent cell damage against ROS. Quercetin as positive control demonstrated IC_50_ of 9.1 ± 0.42 µM.

#### TPC activity

The total phenolics of the extract were determined spectrophotometrically according to the Folin–Ciocalteau procedure. The Folin–Ciocalteau assay relies on the transfer of electrons in alkaline solution from phenolic compounds to phosphomolybdic/phosphotungstic acid complexes. Total polyphenol content was 252.67 ± 2.71 mg GAE/g of dry extract.

### Effects of different treatments on body weight

The difference in body weight average on the 1^st^ and 14^th^ days are demonstrated in Table.1. Wound-induced rats showed significant weight loss compared to the control group (no induced wound). The rats treated by hydroalcoholic extract of the guajava leaves showed remarkable bodyweight improvement.

**Table 1:** Bodyweight changes (g) during the experiment among different groups on the 1^st^ and 14.^th^ days

**Group**	**Bodyweight changes**
1: control group (no induced wound)	27.5 ± 3.3
2: wound + no intervention	-12.5 ± 2.2
3: wound + normal saline (W + N.S), 0.9% mouthwash	19.0 ± 2.5
4: wound + phenytoin suspension (W + P), 30 mg /5 ml mouthwash	25.8 ± 4.1
5: wound + hydroalcoholic extract of the guajava leaves (W + G), 10% mouthwash	24.3 ± 2.8

Data are expressed as Mean ± SD

### Biochemical analyses

#### IL-6 assessment

**Fig. 3:**
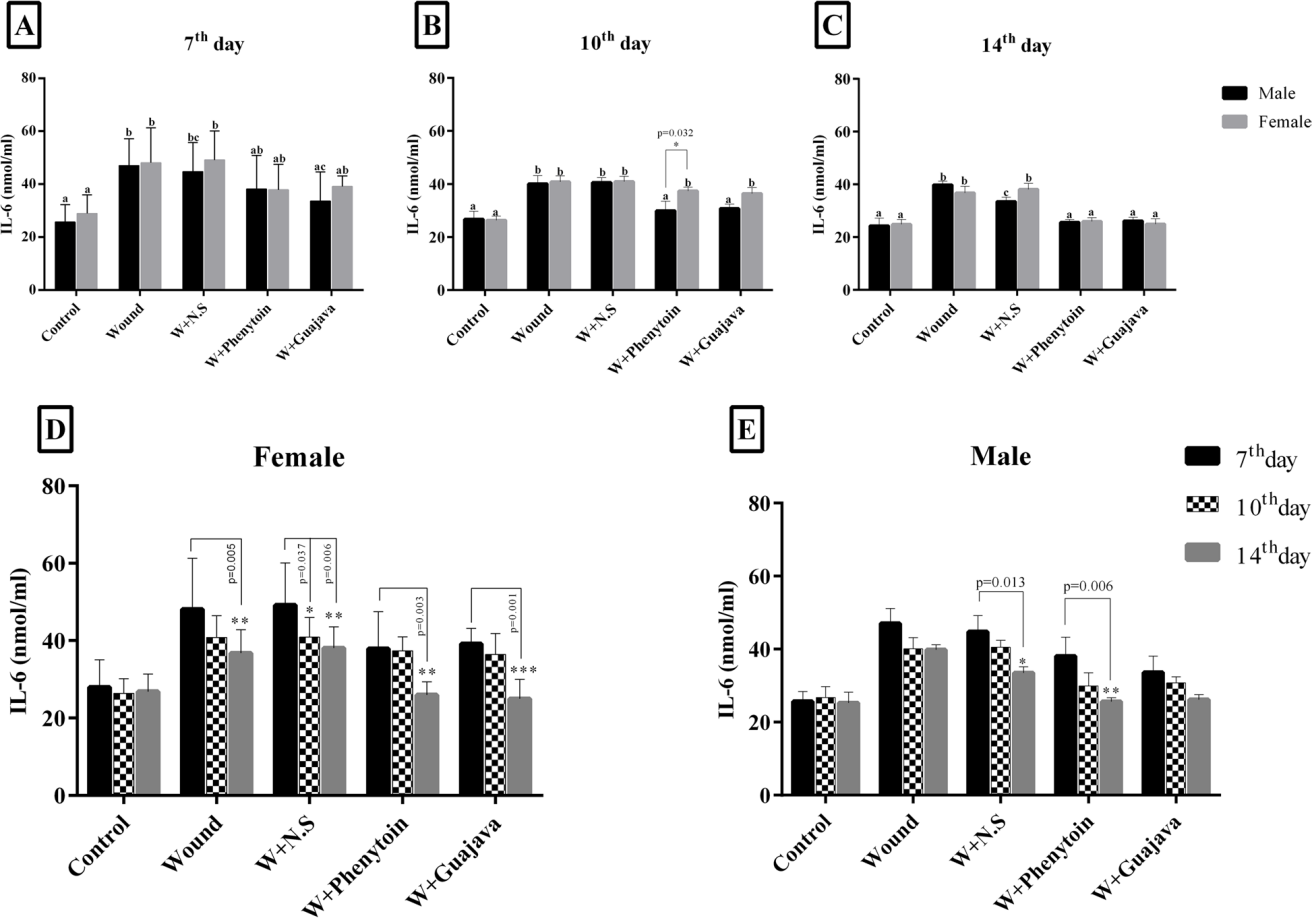
Graphical illustration of IL-6 changes in female and male rats during the intervention on 7^th^ (**A**), 10^th^ (**B**), and 14^th^ days (**C**) as well as IL-6 changes in female rats on 7^th^, 10^th^, and 14^th^ days (**D**) and IL-6 changes in male rats on 7^th^, 10^th^, and 14^th^ days (**E**). Dissimilar letters indicate a significant difference (*p* < 0.05). The number of animals in each group = 12 for 7^th^ & 10^th^ days, The number of animals in each group = 10 for 14^th^ day. According to the posthoc Tukey test which was used for comparison between 7^th^, 10^th^, and 14.^th^ days, in which * was representative for *p* < *0.05*, ** was representative for *p* < *0.01* and *** was representative for *p* < *0.001*

The effects of treatments on IL-6 are shown in Fig. 3. IL-6 concentration was increased in the wound group significantly (*p*-value < 0.001), (Fig. 3A, B and C). A decreasing trend in the amount of IL-6 serum of both treated groups was seen after the 7^th^ day. However, on the 14^th^ day of the study, more reduction was seen in the guajava and phenytoin treatment groups compared with the wound and W + N.S groups (Fig. 3C). There were no significant differences between the male and female rats on the 7^th^ and 14^th^ days (Fig. 3A, C). However, only after 10 days, significant differences between male and female rats in the phenytoin receiving group were seen (*p*-value = 0.032) (Fig. 3B). By the end of the experiment, no significant difference was seen between the phenytoin treatment groups as positive control and the guajava treated one (Fig. 3C). IL6 concentration of female rats in all groups (except the control group) on the 14^th^ day showed a significant decrease compared to the 7^th^ day (*p* < 0.01). Also, the IL6 concentration of female rats on the 10^th^ day in the W + N.S group showed a significant decrease compared to the 7^th^ day (*p* = 0.037). (Fig. 3D). In phenytoin and W + N.S groups, the mean IL6 concentration of male rats on the 14^th^ day showed a significant decrease compared to the 7^th^ day (*p* < 0.05) (Fig. 3E).

#### TAC results

**Fig. 4:**
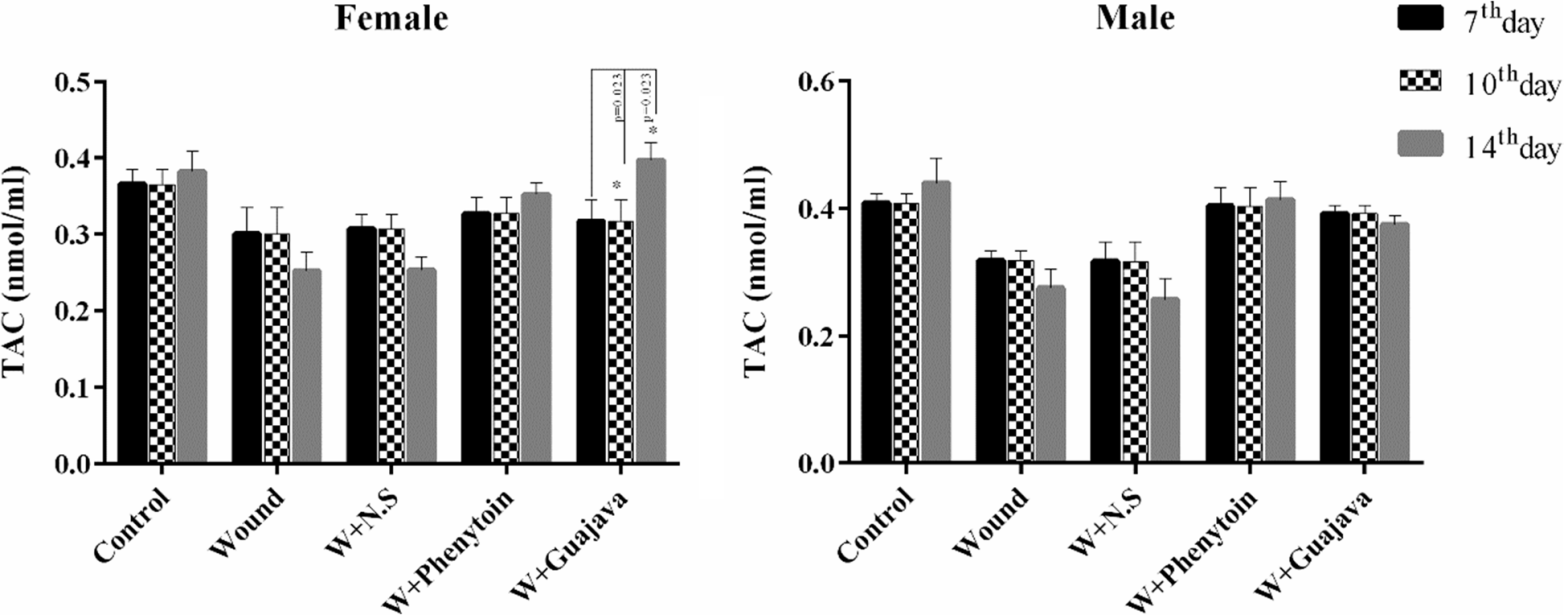
Graphical illustration of TAC changes in female and male rats during the intervention (The number of animals in each group = 6 for 7^th^ & 10^th^ days, The number of animals in each group = 5 for 14^th^ day). According to the posthoc Tukey test which was used for comparison between 7^th^, 10^th^, and 14.^th^ days, in which * was representative for *p* < *0.05*

No statistically significant changes were seen in the TAC outcomes between each specific intervention group during the study period in male rats (Fig. 4). There were no significant differences between male and female rats after the 7^th^ and 14^th^ days in TAC. However, only after 10 days, marginally significant differences between the male and female rats in the phenytoin receiving group were seen for TAC (*p*-value = 0.028). At the end of the study, higher levels of TAC in the guajava and phenytoin treatment groups were seen and this increase was more for the guajava group in female rats.

### Histopathological evaluation

**Fig. 5:**
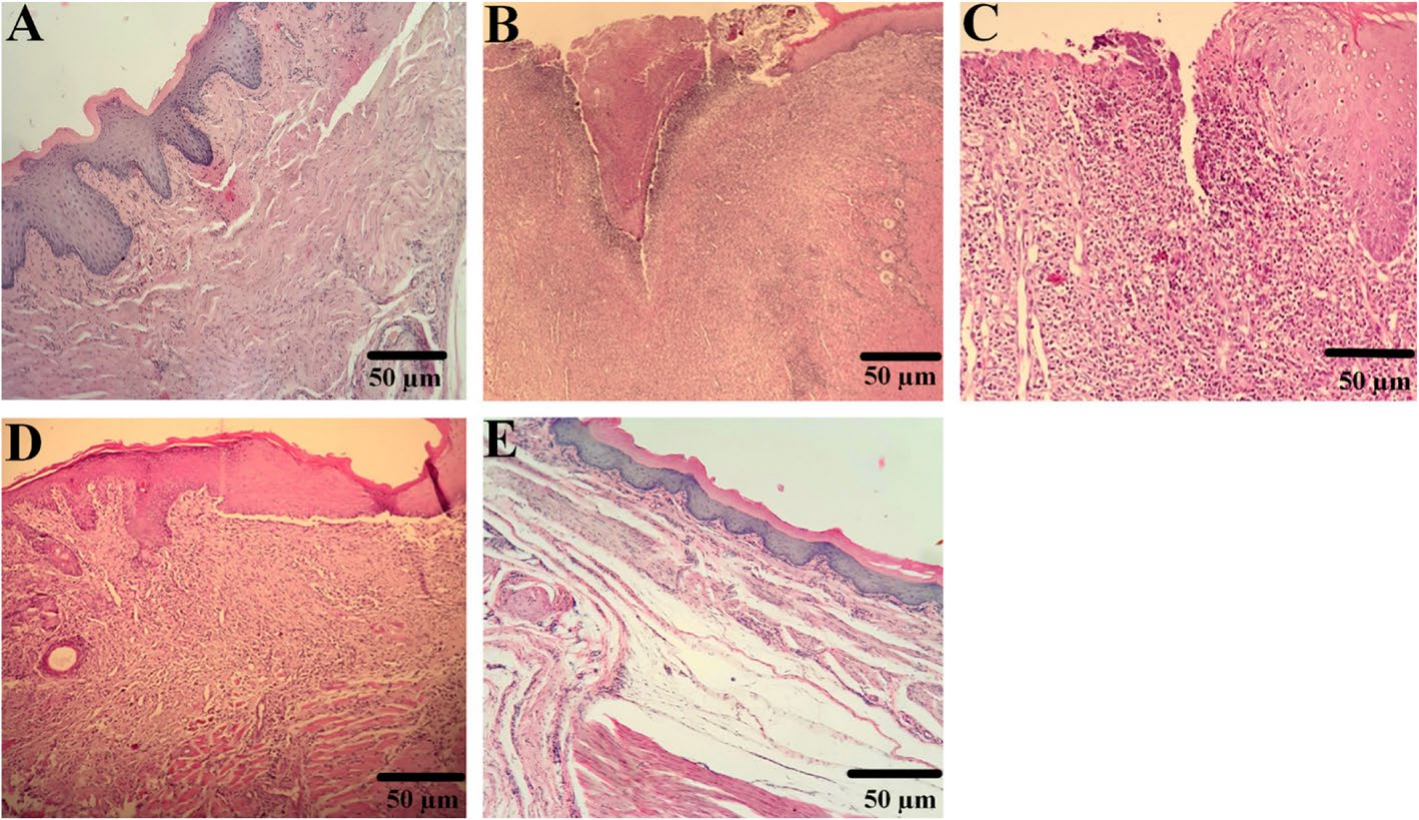
Histopathological features at 14 days post‐wounding H&E staining with magnification at × 100. **A** Healthy control rats with normal tissue structure, **B** wound group with an increment in Deep and extensive ulceration, necrosis of cells and sloughing (shedding) of necrotic and inflammatory cells, severe polymorphonuclear infiltration, and granulation tissue formation. **C** W + N.S group with increment ulceration, granulation tissue formation, with acute and chronic inflammatory infiltrate. **D**, **E** oral mucosal wound rats treated with phenytoin and guajava extract showed Re-epithelialization, late granulation tissue (fibroblastic proliferation), and little mononuclear inflammatories infiltrate

**Fig. 6:**
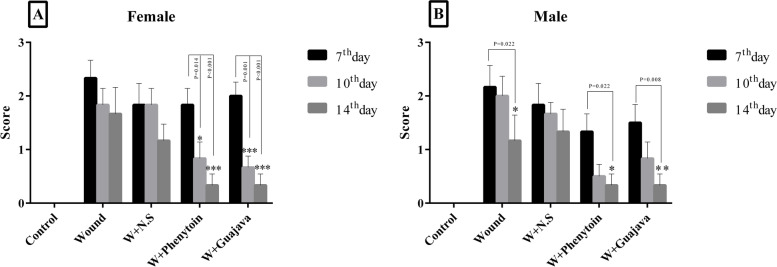
Histopathological total changes in male and female rats (The number of animals in each group = 6 for 7^th^ & 10^th^ days, The number of animals in each group = 5 for 14^th^ day). According to the posthoc Tukey test which was used for comparison between 7^th^, 10^th^, and 14.^th^ days, in which * was representative for *p* < *0.05*, ** was representative for *p* < *0.01* and *** was representative for *p* < *0.001*

Histopathological photos of the buccal mucus tissue are depicted in Fig. 5. According to Fig. 6, no significant differences were seen in histopathologic scoring of the wound after 7 days of interventions in all rats, but on the 10^th^ and 14^th^ days wound score evaluations revealed that guajava and phenytoin treatment decreased the wound mean scores significantly (about 1 point in each evaluation) compared to the wound and W + N.S groups. Overall, it can be understood that the administration of guajava in females (Fig. 6A) and male rats (Fig. 6B) returned the histological parameters to around normal conditions, especially after the14^th^ day without significant differences compared to the W + Phenyton treated group.

### Stereological assessment

**Fig. 7:**
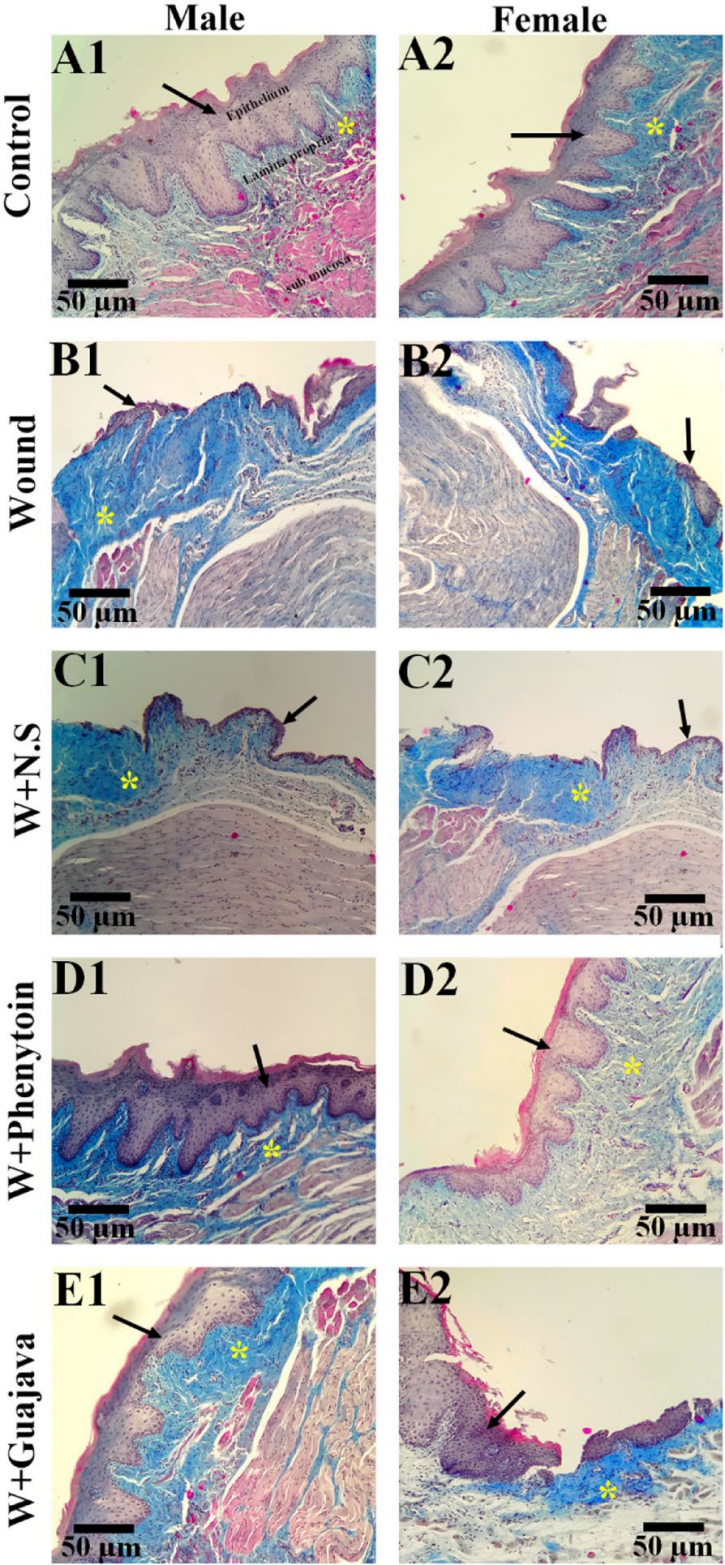
The representative histological image of the wound healing in oral mucosa areas in the experimental groups. Trichrome Masson staining with magnification at × 100. (**A1** and **A2**) Healthy control rats with normal histoarchitecture, (**B1**, **B2**, **C1**, and **C2**) wound and W + N.S groups with degenerative changes, the results showed the wide necrotic and inflamed area, and the black arrow points to the mucosal epithelium. (**D1**, **D2**, **E1**, and **E2**) A representative section of Phenytoin and guajava treated tissues at the end of the study. The black arrow shows complete mucosal epithelial repair and healing. The yellow star indicates the lamina propria layer

Based on the biochemical and histological results, the best biological effects were seen after the 14^th^ day of guajava extract administration. As a result, stereology measurements were monitored after the 14^th^ day of administration. As can be seen in the original tissue section (Fig. 7), a significant decrease in the volume density and thickness of the epithelium and lamina propria layer in the wound and W + N.S g roups, compared to the control group, was observed. Also, a significant reduction in the thickness of the sub-mucosal layer was observed in the wound and W + N.S groups compared to the control group. The epithelium and Lamina propria and submucosa thickness of the phenytoin and guava treated groups demonstrated a significant increase compared to the wounded and W + N.S groups.

#### Effect of guajava extract on epithelial thickness

**Fig. 8:**
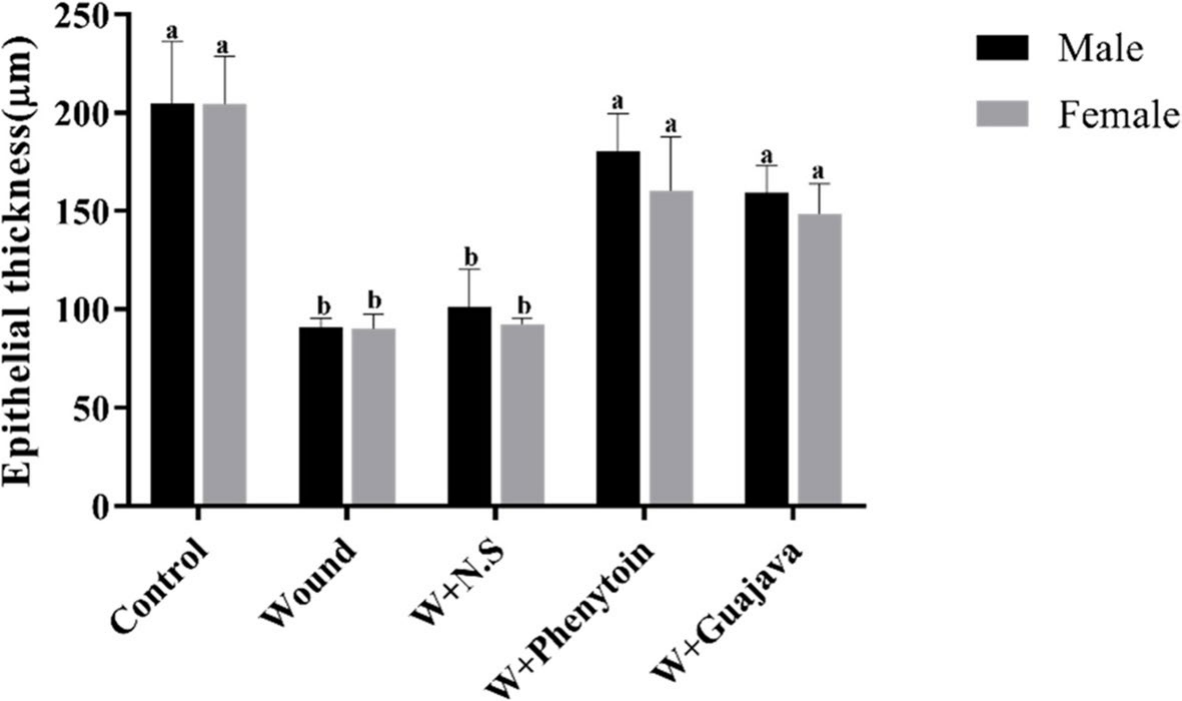
Epithelial thickness comparative graphical illustrations in day 14.^th.^ (The number of animals in each group = 10 (male = 5, female = 5) in each group). According to the posthoc Tukey test which was used for intergroup comparisons, groups with the same superscripted letters were not significantly different at α = 0.05 (*p* ≥ 0.05). However, dissimilar letters indicate a significant difference (*p* < 0.05). The comparison of black columns (males) and gray columns (females) between experimental groups was done separately

As depicted in Fig. 8, after 14 days from the beginning of the study, there were statistically significant differences between the control group (not wounded) and wounded rats with no intervention in epithelial thicknesses. The epithelial thickness of the guajava-treated groups demonstrated a significant increase compared to the wounded group (*ρ* value = 0.008). Comparisons of the guajava-treated group and phenytoin treatment groups revealed no significant differences between both groups in the repaired epithelial thicknesses.

#### Effect of guajava extract on Lamina propria thickness

**Fig.9:**
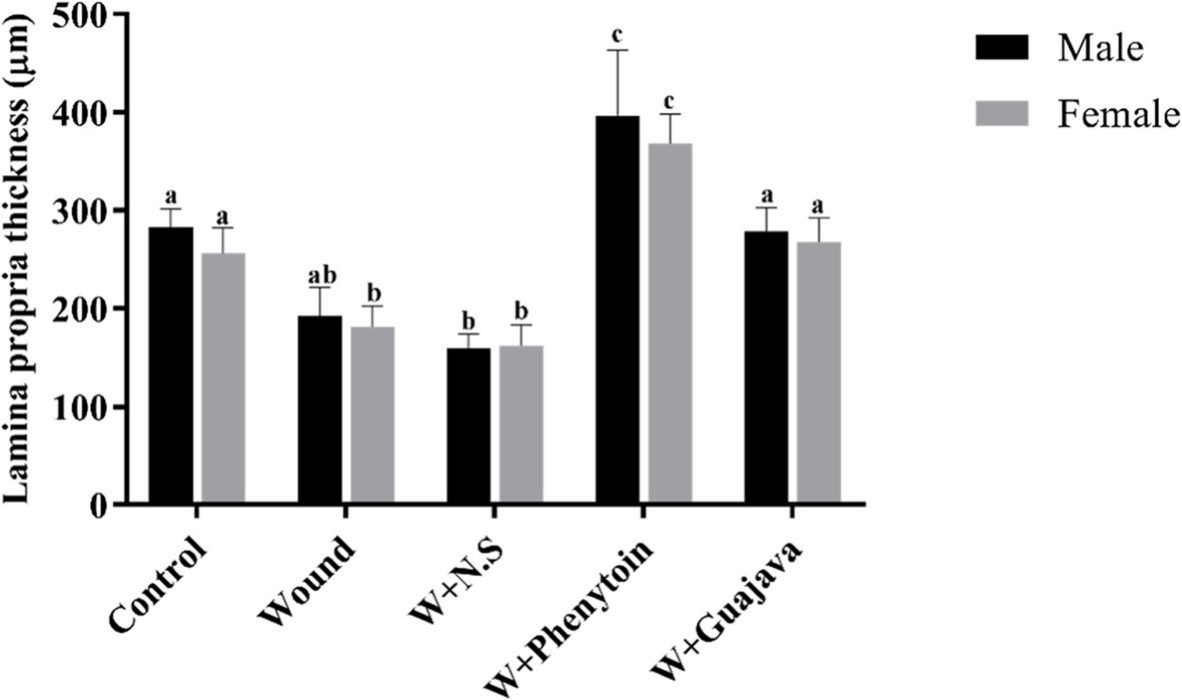
Lamina propria thickness comparative graphical illustrations in day 14.^th^ (The number of animals in each group = 10 (male = 5, female = 5) in each group). According to the posthoc Tukey test which used for intergroup comparisons, groups with the same superscripted letters were not significantly different at α = 0.05 (*p* ≥ 0.05). However, dissimilar letters indicate a significant difference (*p* < 0.05). The comparison of black columns (males) and gray columns (females) between experimental groups was done separately

Similar effects were observed on the lamina propria thickness compared to the epithelial thickness (Fig. 9). The lamina propria thickness was reduced in the wound group; however, the intake of guajava extract reversed it to around normal conditions (no significant differences were seen between control and guajava extract group). The side effect of administration of phenytoin was observed in the treatment group (*ρ* value < 0.001) *through* increase in the thicknesses of the lamina propria significantly even more than this thickness in the no wound rats (control).

#### Effect of guajava extract on sub-mucosal thickness

**Fig. 10:**
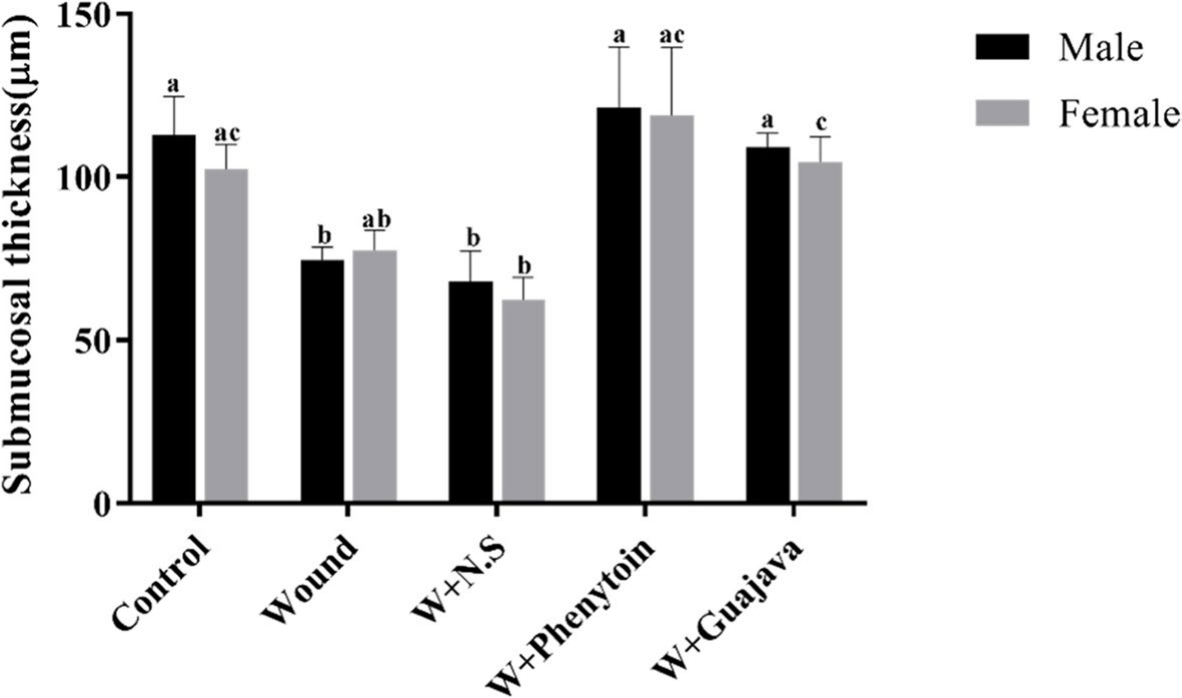
Sub-mucosal thickness comparative graphical illustrations in day 14.^th^ (The number of animals in each group = 10 (male = 5, female = 5) in each group). According to the posthoc Tukey test which was used for intergroup comparisons, groups with same superscripted letters were not significantly different at α = 0.05 (*p* ≥ 0.05). However, dissimilar letters indicate a significant difference (*p* < 0.05). The comparison of black columns (males) and gray columns (females) between experimental groups was done separately

Data in Fig. 10 show that oral mucosal wound in rats decreased the sub-mucosal significantly; the guajavatreated group returned these parameters to around normal conditions.

#### Effect of guajava extract on the number of fibroblasts

**Fig. 11:**
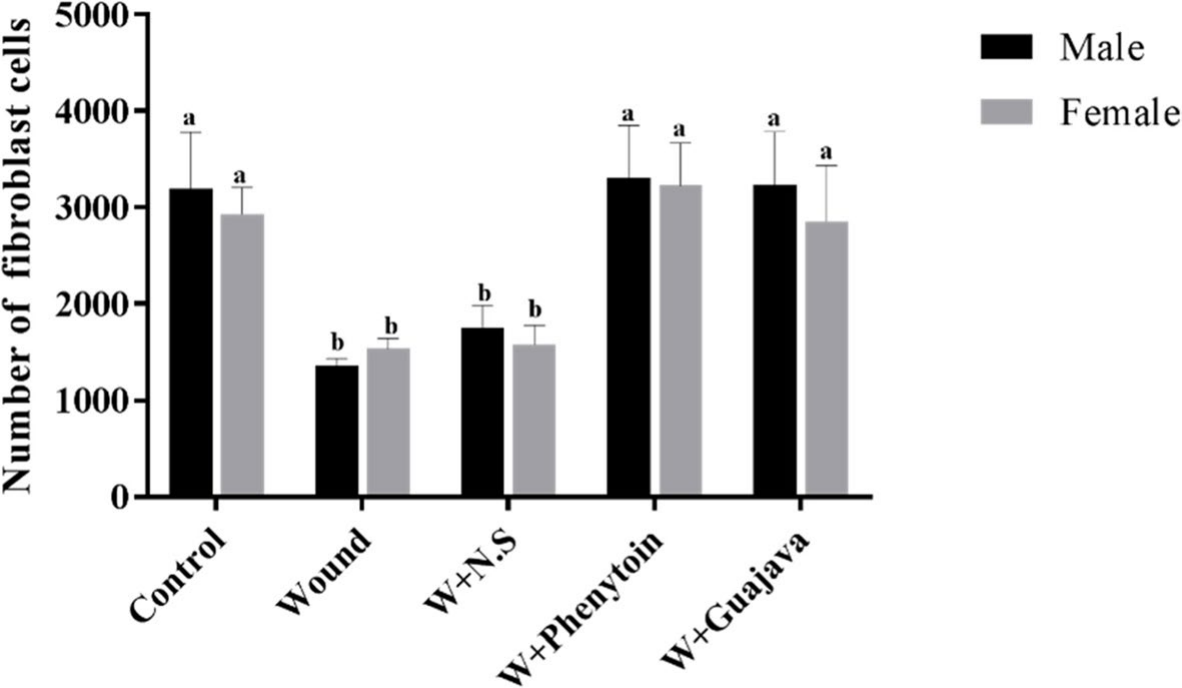
Number of fibroblasts comparative graphical illustrations in day 14.^th^ (The number of animals in each group = 10 (male = 5, female = 5) in each group). According to the posthoc Tukey test which was used for intergroup comparisons, groups with the same superscripted letters were not significantly different at α = 0.05 (*p* ≥ 0.05). However, dissimilar letters indicate a significant difference (*p* < 0.05). The comparison of black columns (males) and gray columns (females) between experimental groups was done separately

**Fig. 12:**
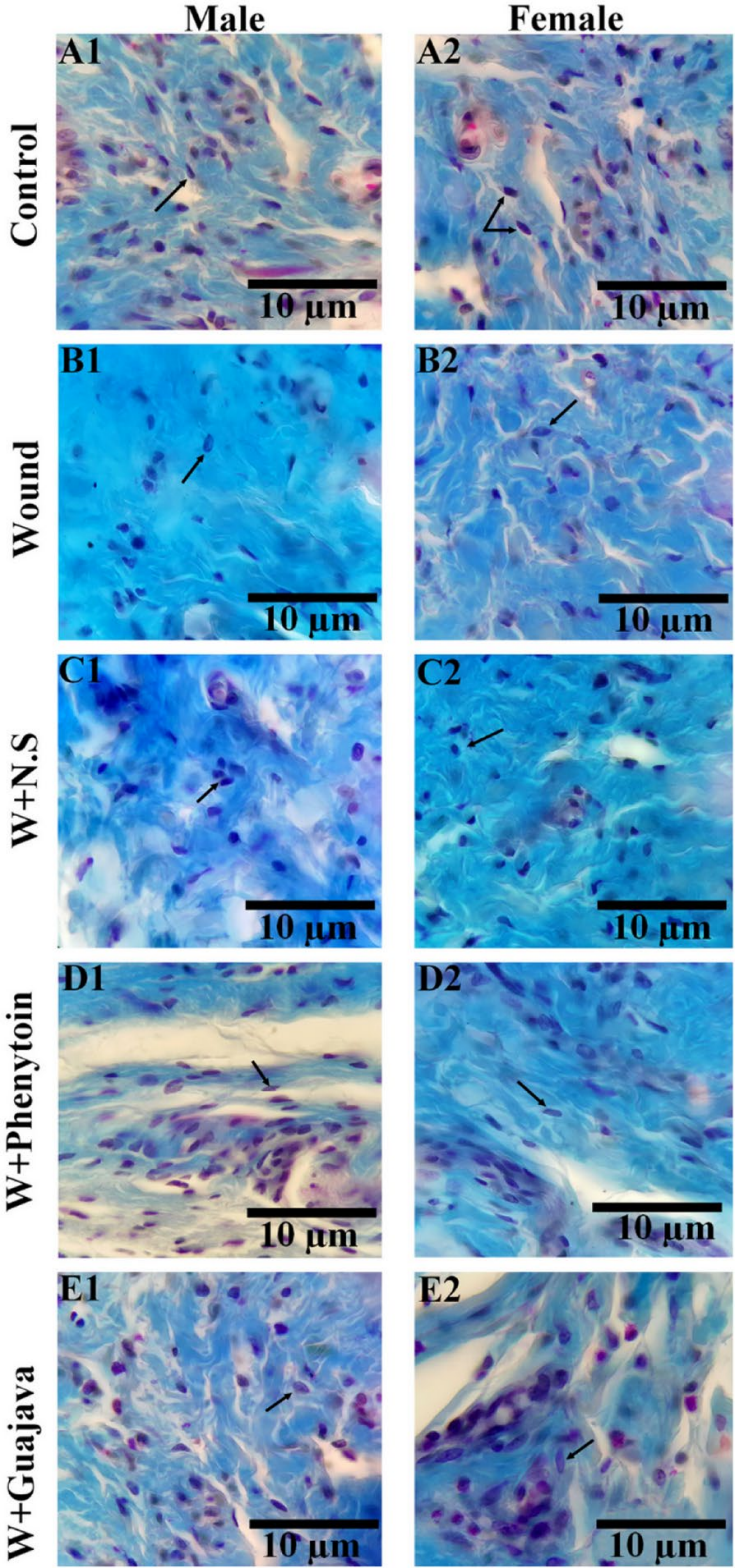
Comparison of the microscopic images of the number of fibroblasts cells in the experimental groups. Trichrome Masson staining with magnification at × 1000. The scale bar is 10 μm

The number of fibroblasts in the experimental groups is shown in Fig. 11. As shown, in rats with oral mucosal wound, a significant decrease was seen in the number of fibroblasts in the wound group compared to the control (*ρ* value = 0.008). Interestingly, the administration of guajava extract increased the number of fibroblasts during the treatment. No significant difference was observed between the guajava-treated rats and the phenytoin-treated rats. Also, the original tissue section depicted fewer fibroblasts cells in the wound and W + N.S rats. Treatment of the wound rats with the phenytoin and extract ameliorated the loss of fibroblast cells. The arrow indicates the fibroblast cell (Fig. 12).

#### Effect of guajava extract on the volume density of the epithelium

**Fig. 13:**
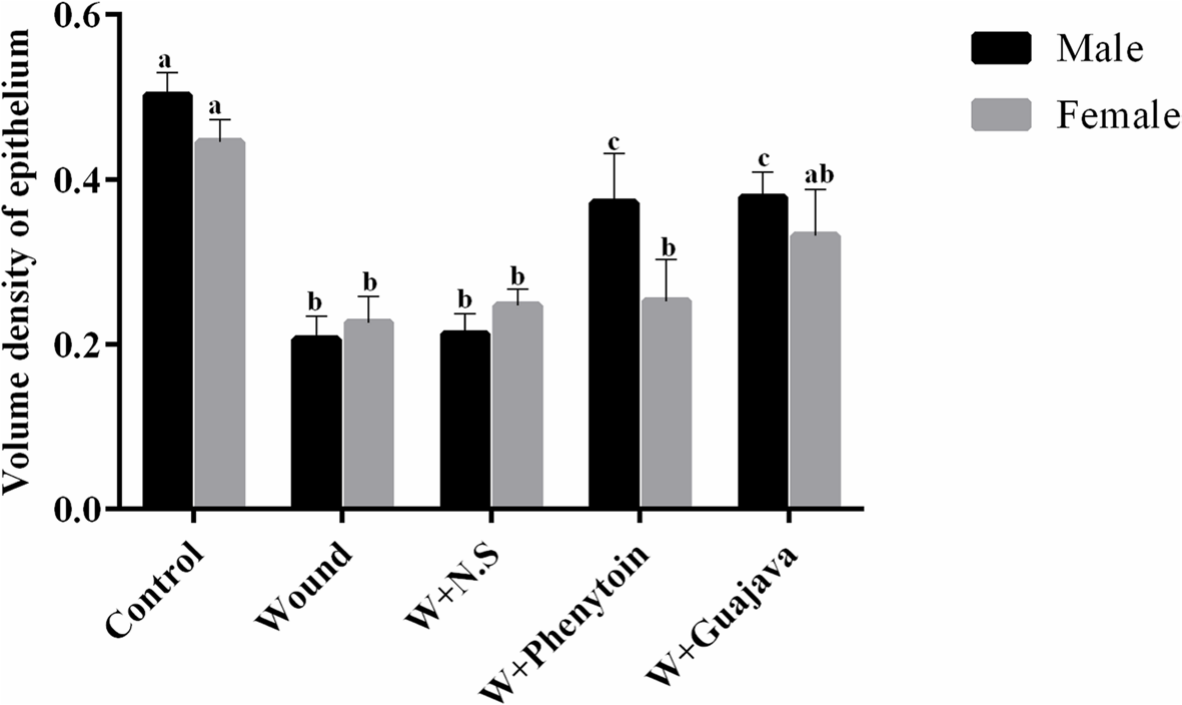
Volume density of epithelium comparative graphical illustrations in day 14.^th^ (The number of animals in each group = 10 (male = 5, female = 5) in each group). According to the posthoc Tukey test which was used for intergroup comparisons, groups with the same superscripted letters were not significantly different at α = 0.05 (*p* ≥ 0.05). However, dissimilar letters indicate a significant difference (*p* < 0.05). The comparison of black columns (males) and gray columns (females) between experimental groups was done separately

It is well known that the volume density of epithelium was reduced in rats by the oral mucosal wound (Fig. 13). A comparison between male and female rats in different groups for volume density of the epithelium changes showed statistically significant different responses based on gender. Almost all wounded female rats in the four groups did not improve the volume density of the epithelium enough to be considered as a healed wound when compared with not-wounded rats. The wounded male rats in the phenytoin and guava treatment groups showed a significantly higher volume density of the epithelium compared to female rats in their similar group, but even male rats of our study did not increase their volume density enough to become the same as the control group after 14 days of interventions.

#### Effect of guajava extract on the volume density of the submucosae

**Fig. 14:**
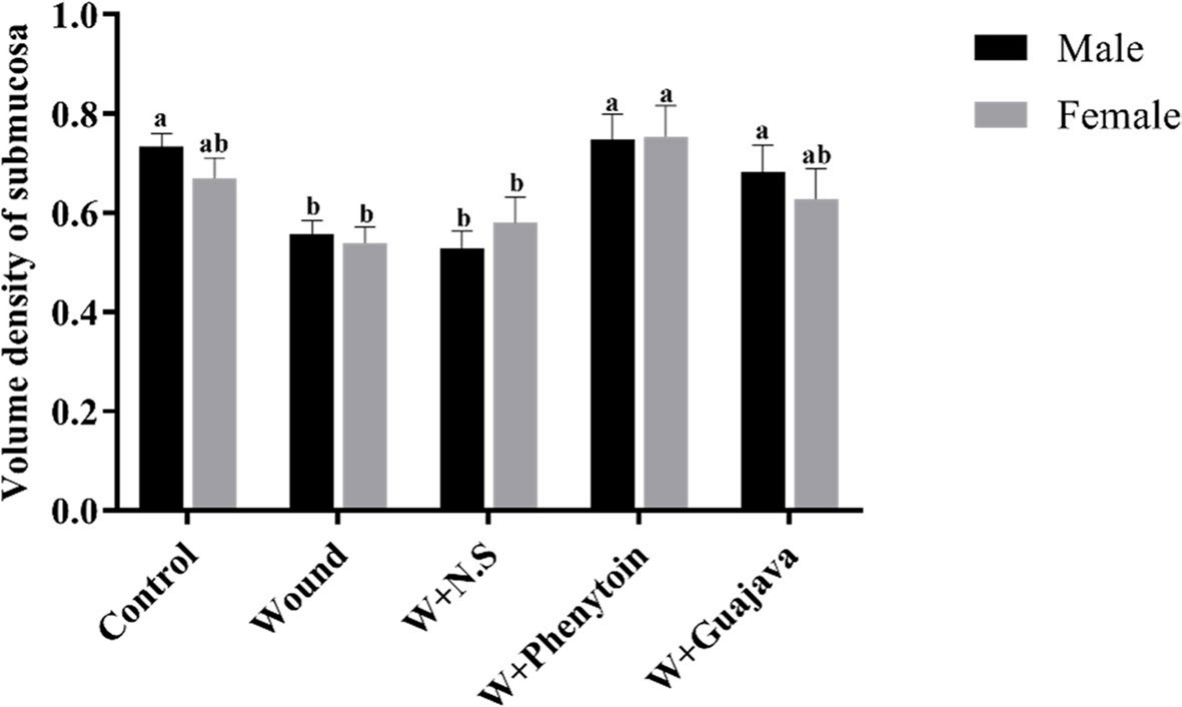
Volume density of submucosae comparative graphical illustrations in day 14.^th^ (The number of animals in each group = 10 (male = 5, female = 5) in each group). According to the posthoc Tukey test which was used for intergroup comparisons, groups with the same superscripted letters were not significantly different at α = 0.05 (*p* ≥ 0.05). However, dissimilar letters indicate a significant difference (*p* < 0.05). The comparison of black columns (males) and gray columns (females) between experimental groups was done separately

As depicted in Fig. 14, the volume density of the submucosae was decreased in the wound group compared to the control one. The rats in the guajava and phenytoin treatment groups could increase enough volume density to be considered as a healed sub-mucosae layer.

## Discussion

In this study, the effect of hydroalcoholic extract of guajava leaves on oral mucositis induced in rats was investigated and evaluated through phytochemical, biochemical, histopathologic and stereological studies. OM is a frequent inflammatory complication of cancer chemo- or radiotherapy that is characterized by pain, erythema, and the formation of ulcers [1]. OM happened due to direct damage to DNA via reactive oxygen and nitrogen species, causing cell damage in the epithelium and subepithelial mucosa. Next, transcription factors are stimulated and the production of proinflammatory cytokines, such as TNF-α, IL-1 and IL-6 increased. Subsequently, the cytotoxic agents reduce the mitosis of dividing the epithelial cells in the oral cavity causing atrophy and ulceration [43–45]. Currently, OM treatment is based on symptomatic care due to the lack of effective treatments to manage OM complications [2].

*Psidium guajava L.* is an inexpensive fruit in tropical areas. Different parts of the plant are used in traditional medicine for the treatment of various human ailments such as wounds, ulcers, bronchitis, and diarrhea [46]. The main biochemical components of *Psidium guajava L* leaves are flavonoids, monoterpene, diterpene, triterpenoids, alkaloids, tannins, vitamins (especially vitamin C and vitamin A.), and enzymes [47, 48]. Quercetin was isolated and identified as the major active compound in leaves [47]. Also, guayavolic acids, avicularin, asiatic acid, luteolin, guajavolide and kaempferol [49, 50], as well as two triterpenoids, guavanoic acid and guavacoumaric acid, were identified from the leaves of *Psidium guajava* [51]. Another study also reported the major contents present in the extract of 100 g dried leaf *Psidium guajava* were catechin (846 mgr), gallic acid (681 mgr), and resveratrol (71 mgr) [52]. The anti-inflammatory and anti-tumor effects of this plant could be attributed to these components. IL-6 acts as a pro-inflammatory cytokine that is secreted by T cells and macrophages to stimulate an immune response [47]. Our results revealed a decreasing trend in the amount of IL-6 in the serum of all wounded rats significantly in the guajava and phenytoin treatment groups compared with the wound and W + N.S groups. These findings indicated that treatments with phenytoin and guajava leaves extract resulted in a more and quicker reduction in wound-induced IL-6 level to the base level before wounding (like control group level) during the healing process.

An increase in the oxidative enzyme activity and/or reduction in the activity of the antioxidative enzymes cause oxidative stress and this situation leads to cell damage, non-wound healing in the pathogenesis of chronic, premature aging and even neoplastic transformation. Therefore, ROS balance is essential for the normal repair process [53]. Under the physiologic conditions, living organisms have developed antioxidant systems, macromolecules and small molecules antioxidant, so that the formation and elimination of ROS are in balance [54]. The sum of endogenous and food-derived antioxidants represents the total antioxidant activity of the extracellular fluid to inactivate ROS and their damage. TPC was usually related to the prevention of inflammation and degenerative diseases. TPC was performed to determine the amounts of phenolic compounds as powerful antioxidants in the guajava leaves extract. In this study, the DPPH assay, as a popular and reliable antioxidants detection method, was used for the evaluation of the free radical scavenging potential of the guajava extract. Guajava leaves extract possessed significant antioxidant and free radical scavenger activities and is a potential source of natural antioxidants.

These antioxidant properties of the guajava leaf are associated with its phenolic compounds such as gallic acid, ellagic acid, ascorbic acid, quercetin, and resveratrol [12]. Many studies revealed the beneficial effects of gallic acid in wound healing. Alves Barros et al. in 2017 indicated that topical applications of gallic acid had wound healing activity when administered in wounded rats since its pharmacological action was associated with a possible topical anti-oxidant and anti-inflammatory activities [55]. Flavonoids contributes to increasing collagen synthesis which promotes cross-linking of collagen, decreasing the degradation of soluble collagen, promoting the conversion of soluble collagen to insoluble collagen, and hindering the catabolism of soluble collagen [49]. Anthocyanins are a group of dietary bioactive flavonoids found in the guajava leaves. Anthocyanins exert potent antioxidant, anti-inflammatory and wound healing activity through inhibition of cyclooxygenase and transformation of malignant oral tissues [13]. Resveratrol's antioxidant properties were demonstrated by its ability to directly scavenge free radicals, lower MDA levels in lipid peroxidation products, and increase the activity of antioxidant enzymes such as GPx and SOD [50, 51]. Kim et al. (2015) also showed that ursolic acid isolated from the guajava leaves inhibited the inflammatory mediators and ROS in stimulated macrophages, and that it could improve the blood flow by modulating nitric oxide (NO) secretion from vessels endothelial cells, which is necessary for optimal wound healing [56]. In their study, Yuniarti et al. (2018) discovered that applying ellagic acid topically for 14 days to albino rats' incised wounds accelerated wound healing by improving collagen deposition, polymorphonuclear neutrophil infiltration in the wound area, angiogenesis, and fibrosis degree [57]. Studies demonstrated the significant anti-inflammatory activity of the guajava leaves against acute, sub-acute, and chronic inflammation was due to its flavonoids compounds, in particular quercetin that led to the lower release of IL-6 [58]. Tanideh et al. (2017) in their study showed that hydro-ethanolic extracts of the guajava leaf appear to prevent osteoarthritis by inhibition of free radical formation in the knee joint of guinea pig [59]. Chah et al. (2006) also determined the wound healing properties of a methanolic leaf extract of *Psidium guajava* using the excision wound model and observed more than 90% wound healing after 14 days post-surgery [60].

TAC was used to measure the total antioxidant capacity of the plasma and cell lysates [61]. At the end of this study, higher levels of TAC in the guajava and phenytoin treated groups were seen when compared with decreased levels in the groups with no wound. Moreover, this increase was seen more in the guajava group in female rats. Our findings were similar to those of Rajamanickam et al. (2013) who evaluated antibacterial and wound healing activities of quercetin-3-O-A-L-rhamnopyranosy, showing that the quercetin ointment (1%) alone and in combination with quercetin orally resulted in a significant increase in wound contraction by enhanced epithelization [62].

Epidemiologic studies have previously reported controversial results about the impact of sex on wound repair. One critical mediator of wound healing is the androgens hormone, which accelerates repair in both human and animal models [63]. In an interesting study performed by João Paulo Steffens et al. (2018), the role of androgens on periodontal repair in female rats was assessed; it was concluded that testosterone administration decreased the levels of proinflammatory cytokines like IL-4, IL-1 and IL-6. It means that higher amounts of male hormones suppress the release of proinflammatory cytokines (like IL-6). The result of the present study, in agreement with previous studies, revealed that female rats had a higher serum status of IL-6. On the 10^th^ day, the decreasing trend of IL-6 in male rats (more androgens) which received phenytoin was more than female rats. Our interventions improved the volume density of the epithelium and made the healing process shorter. Although wounded male rats in the phenytoin and guajava treatment groups showed a significantly higher volume density of the epithelium compared to female rats in their same group. Mariotti et al. (2013) evaluated the endocrinology of sex steroid hormones and cell dynamics in the periodontium and concluded specific populations of the fibroblasts and epithelial cells would be modulated by sex steroid hormones [64]. The results of our study indicate that the hydroalcoholic extract of guajava repaired the lamina propria membrane effectively to the same thickness as before wounding. However, phenytoin over-repaired the lamina propria layer to a significantly higher thickness from before wounding which might be due to stimulation of fibroblast proliferation [65]. Reduction in the wound’s pathological scores means that the wound is healing. The histopathological outcomes in the present study revealed well-repaired tissue layers, an increase in fibroblast cells, and finally, epithelium formation after the wound was treated with phenytoin and guajava leaves. We also demonstrated that after 14 days both of our interventions could repair the sub-mucosal layer, and increased the number of fibroblast cells and volume density of the sub-mucosae to the same thickness to be considered as healed sub-mucosae layer. However, further research is needed on herbal medicines and their doses and regimens to demonstrate their superiority over chemical drugs.

## Conclusions

Regarding natural and homeopathic agents, the application of guajava leaves hydroalcoholic extract as a mouthwash, patch, syrup, or topical product with anti-inflammatory and anti-oxidant properties on oral wounds and ulcers may result in fast epithelialization from the biochemical and histopathological points of view which contribute to tissue healing.

## Supplementary Information

**Additional file 1: Figure S1.** Graphical illustration of IL-6 changes in Female and Male rats during the intervention.

## Data Availability

The datasets used and/or analysed during the current study are available from the corresponding author on reasonable request.

## Abbreviations

OM: Oral mucositis
TPC: Total phenolic compound
GPx: Glutathione peroxidase:
SOD: Superoxide dismutase:
MDA: Malondialdehyde
ROS: Reactive oxygen species
NO: Nitric oxide
IL-6: Interleukin-6
iNOS: Nitric oxide synthase
COX-2: Cyclooxygenase-2
NF-κB: Nuclear factor-kappa B
