# Supplementary material for: The effect of hydroalcoholic extract of Psidium guajava L. on experimentally induced oral mucosal wound in rat
Source: BMC Complement Med Ther. 2022 Jul 29;22:201. doi: 10.1186/s12906-022-03655-5 (PMC9338486; doi:10.1186/s12906-022-03655-5)
Supplement: Supplementary file 1 — Additional file 1: Figure S1. Graphical illustration of IL-6 changes in Female and Male rats during the intervention. [file 12906_2022_3655_MOESM1_ESM.docx]

**

**

**

**

**

**

**Fig. S1.** Graphical illustration of IL-6 changes in Female and Male rats during the intervention

(N=6 for 7^th^& 10^th^, N= 5 for 14^th^ in each group). According to the posthoc Tukey test which was used for intergroup comparisons, groups with the same superscripted letters were not significantly different at α= 0.05 (p≥0.05). However, dissimilar letters indicate a significant difference (p<0.05).
